# Changes in cardiac structure and function from 3 to 12 months after hospitalization for COVID‐19

**DOI:** 10.1002/clc.23891

**Published:** 2022-08-03

**Authors:** Tarjei Øvrebotten, Peder Myhre, Jostein Grimsmo, Albulena Mecinaj, Divna Trebinjac, Magnus B. Nossen, Simon Andrup, Tony Josefsen, Gunnar Einvik, Knut Stavem, Torbjørn Omland, Charlotte B. Ingul

**Affiliations:** ^1^ Department of Cardiology, Division of Medicine Akershus University Hospital Lørenskog Norway; ^2^ Institute of Clinical Medicine University of Oslo Oslo Norway; ^3^ LHL Hospital Gardermoen Jessheim Norway; ^4^ Department of Cardiology Østfold Hospital Trust Kalnes Grålum Norway; ^5^ Pulmonary Department Akershus University Hospital Lørenskog Norway; ^6^ Health Services Research Unit Akershus University Hospital Lørenskog Norway; ^7^ Department of Circulation and Medical Imaging Norwegian University of Science and Technology Trondheim Norway

**Keywords:** COVID‐19, echocardiography, postacute COVID syndrome

## Abstract

**Background:**

Cardiac function may be impaired during and early after hospitalization for COVID‐19, but little is known about the progression of cardiac dysfunction and the association with postacute COVID syndrome (PACS).

**Methods:**

In a multicenter prospective cohort study, patients who had been hospitalized with COVID‐19 were enrolled and comprehensive echocardiography was performed 3 and 12 months after discharge. Twenty‐four‐hour electrocardiogram (ECG) was performed at 3 and 12 months in patients with arrhythmias at 3 months.

**Results:**

In total, 182 participants attended the 3 and 12 months visits (age 58 ± 14 years, 59% male, body mass index 28.2 ± 4.2 kg/m^2^). Of these, 35 (20%) had severe COVID‐19 (treatment in the intensive care unit) and 74 (52%) had self‐reported dyspnea at 3 months. From 3 to 12 months there were no significant overall changes in any measures of left or right ventricle (LV; RV) structure and function (*p* > .05 for all), including RV strain (from 26.2 ± 3.9% to 26.5 ± 3.1%, *p* = .29) and LV global longitudinal strain (from 19.2 ± 2.3% to 19.3 ± 2.3%, *p* = .64). Changes in echocardiographic parameters from 3 to 12 months did not differ by COVID‐19 severity or by the presence of persistent dyspnea (*p* > .05 for all). Among patients with arrhythmia at 3 months, there was no significant change in arrhythmia burden to 12 months.

**Conclusion:**

Following COVID‐19, cardiac structure and function remained unchanged from 3 to 12 months after the index hospitalization, irrespective of COVID‐19 severity and presence of persistent dyspnea. These results suggest that progression of cardiac dysfunction after COVID‐19 is rare and unlikely to play an important role in PACS.

## 1 INTRODUCTION

Cardiac injury, defined by elevated levels of cardiac troponin (cTn) is common in patients hospitalized with COVID‐19^1^ and is associated with severe illness and in‐hospital death.^2^, ^3^, ^4^ Studies assessing cardiac function by echocardiography in hospitalized patients with COVID‐19 have demonstrated impairments in right ventricular (RV) function ^5^, ^6^, ^7^, ^8^ and to a lesser extent left ventricular (LV) dysfunction.^6^ The presence of cardiac dysfunction in the acute phase of COVID‐19 is associated with worse short‐term outcomes.^6^, ^9^, ^10^ In the early convalescent phase, studies have reported inconsistent findings depending on the imaging modality used, the severity of COVID‐19, and the time from the acute infection. Studies using cardiac magnetic resonance (CMR) imaging have suggested that a high proportion of patients have a myocardial scar and diffuse fibrosis,^11^ while others report substantially lower prevalence.^12^ However, the presence of structural and functional cardiac abnormalities in the early convalescent phase varies between echocardiographic studies. A high prevalence of diastolic dysfunction and prevailing reduced RV function but sustained LV function has been shown,^13^, ^14^, ^15^ while others have found little pathology by echocardiography approximately 3 months after hospitalization.^16^, ^17^ Cardiac arrhythmias during acute COVID‐19 have been reported,^18^ while the prevalence and progression of cardiac arrhythmias in the convalescent phase have not been extensively studied.

Although short‐term alterations in cardiac structure and function after COVID‐19 have been described, no study has evaluated longitudinal changes in cardiac structure and function during the first year after hospitalization for COVID‐19. We hypothesized that there would be no significant changes in cardiac structure or function or arrhythmic events from 3 to 12 months after COVID‐19. In this multicenter prospective cohort follow‐up study of patients hospitalized with COVID‐19, we aimed to assess the prevalence of cardiac dysfunction and arrhythmias 12 months after the acute infection, the changes in echocardiographic parameters, and arrhythmic events from 3 to 12 months, according to the severity of the COVID‐19 infection and presence of post‐COVID‐19 dyspnea.

## 2 METHODS

### 2.1 Study population and design

Unselected patients hospitalized for COVID‐19 between February 2020 and June 2020 were included in the Patient‐Related Outcomes and Lung Function after Hospitalization for COVID‐19 (PROLUN) study. PROLUN is a multicenter prospective cohort study at six major hospitals in Norway: Akershus University Hospital, Haukeland University Hospital, Oslo University Hospital Ullevål, and Rikshospitalet, St. Olav's University Hospital, and Østfold Hospital Kalnes.^19^ Patients ≥ 18 years of age who had been admitted for >8 h with a discharge diagnosis of COVID‐19 or viral pneumonia combined with a positive SARS‐CoV‐2 PCR‐test were considered eligible. Patients residing outside the catchment areas of the hospitals, inability to provide informed consent or participation in the World Health Organization Solidarity trial were excluded. Patients willing to participate were invited to follow‐up visits approximately 3 and 12 months after discharge from the index hospitalization for COVID‐19; June 1 to August 28, 2020 and February 26 to June 29, 2021, respectively (Supporting Information: Figure 1). The two study visits were done according to the same prespecified protocols.

Informed consent was obtained by returning a written signed consent form or through a secure digital consent form (Services for Sensitive Data, TSD, University of Oslo). The study was approved by the Regional Ethics Committee for South‐Eastern Norway (#125384), by data protection officers at each participating center and registered in the ClinicalTrials.gov database (NCT04535154). All collected data were stored in Services for Sensitive Data (TSD; University of Oslo), designed for storing and postprocessing sensitive data in compliance with the Norwegian Personal Data Act and Health Research Act.

#### 2.1.1 Echocardiography

Five of the six hospitals in the PROLUN study were part of the echocardiography study, that is, patients from Haukeland University Hospital did not have an echocardiogram or a 24‐h ECG and were therefore not included in these analyses. Transthoracic two‐dimensional echocardiography of patients was performed by five experienced operators according to standard guideline‐recommended methodology using commercially available ultrasound systems (Vivid E 95 GE Horten). LV global longitudinal strain (GLS) and right ventricular free wall strain (RVLS) are expressed as absolute values. Details about the echocardiography methods are presented in Supporting Information Material.

LV hypertrophy was defined as left ventricular mass index (LVMi) > 115 g/m^2^ for men and >95 g/m^2^ for women.^20^ Pathologic LV GLS was defined as <18%, and reduced LV ejection fraction (LVEF) was defined as <55%.^21^ Diastolic dysfunction was defined as ≥2 of 4: Indexed left atrial (LA) volume ≥34 ml/m^2^, lateral E' < 10 cm/s or septal E' < 7 cm/s, tricuspid regurgitation velocity (TRVmax) > 2.8 m/s and elevated LV fillings pressure (defined as E/e' > 14). RV dilatation was defined as RV basal diameter > 41 mm.^20^ Tricuspid annular plane systolic excursion (TAPSE) < 1.8 cm and RVLS < 20% was defined as pathological for RV function.^20^ TRVmax and right atrial pressure, derived from inferior vena cava dimension and collapsibility, was used to calculate systolic pulmonary artery pressure (sPAP). sPAP > 35 was defined as pathological.

#### 2.1.2 Twenty‐four‐hour electrocardiogram (ECG)

Twenty‐four‐hour ECG was performed in 201 patients at the 3‐month visit,^15^ and this was repeated at the 12‐month visit in 40 of the participants (20%) who had pathological findings at 3 months, defined as ventricular tachycardia (VT, nonsustained or sustained), premature ventricular contractions (PVC) > 10%/24 h, atrial fibrillation/flutter, atrioventricular block type 2 or 3, sinoatrial block exceeding 3 s, supraventricular tachycardia exceeding 30 s and extreme sinus bradycardia with <30 beats/minute. The 24‐h ECG was obtained using Schiller Medilog FD12 Plus and Philips DigiTrak XT, and analyzed by a trained cardiologist.

#### 2.1.3 Assessment of dyspnea

Dyspnea was classified by the modified Medical Research Council (mMRC) dyspnea scale at the 3 and 12‐month visits. mMRC is a self‐rating tool to measure the degree of disability that breathlessness poses on day‐to‐day activities on a scale from 0 (no dyspnea) to 4 (maximum dyspnea).^22^ Dyspnea was defined as mMRC ≥ 1 at the 3‐month visit.

### 2.2 Grading of severity

To compare patients based on severity of COVID‐19, we classified patients based on: (1) need for intensive care unit (ICU) treatment compared to medical ward only; (2) respiratory criteria on admission to classify patients as “severe respiratory state” (either without Spo_2_ < 90% without oxygen, SpO_2_ < 95% with oxygen, requiring >3 L O_2_ to maintain SpO_2_ > 95% or respiratory rate > 30/min); (3) peak levels of CRP during the hospitalization to classify patients as “severe inflammatory state” (above the median value).

### 2.3 Statistical methods

The baseline characteristics are presented as mean ± standard deviation or medians (1st to 3rd quartile, Q1−Q3) for continuous variables and as absolute numbers and percentages for categorical variables. Delta values for change in echocardiographic variables from 3 to 12 months were generated by subtraction of the first value from the last value. All echocardiographic delta variables were assessed for normal distribution by histograms, Q−Q plots and the Shapiro−Wilk test. Measurements at the 12‐month visit were compared with the 3‐month measurements by paired sample *t*‐tests, and one sample *t*‐tests for delta values to assess the difference from no change. Nonnormally distributed variables were also tested with the Wilcoxon matched‐pairs signed‐rank test. The proportion of patients with LV hypertrophy, LV systolic and diastolic dysfunction, elevated filling pressures, reduced RV function and RV dilatation at 3 months and 12 months were compared by the McNemar's paired proportions test. To assess individual changes from 3 to 12 months we investigated the proportion of patients with a 15% increase or decrease in LV function (LV GLS and LVEF) and RV function (RVLS and TAPSE), by model of longitudinal studies in cardio‐oncology,^23^ and compared this with the McNemar's test. We also report 10% and 20% changes.

Changes in echocardiographic parameters between patients with versus without treatment in the ICU, and between patients with versus without dyspnea at 3 months, were analyzed using multivariable linear regression. To adjust for possible confounders, we included the following a priori selected covariates in all models: age, sex, systolic blood pressure, heart rate, body mass index, and established cardiovascular disease, in addition to the baseline echocardiographic value for each measure (3‐month visit).

All statistical analyses were performed using Stata Software (version 16; Stata Corp.).

## 3 RESULTS

### 3.1 Patient characteristics

Of the 264 patients included in the PROLUN study, 200 patients had available echocardiograms at the 3‐month visit, and among these 178 (89%) also attended the 12‐month visit (Supporting Information: Figure 1). Patients with both 3‐ and 12‐month echocardiograms available were included in this study, and these patients had comparable baseline characteristics to the remaining PROLUN participants (Supporting Information: Table 1). The 3‐month visit was conducted after a median of 102 (range 70–172) days from hospital discharge and the 12‐month visit after a median of 387 (range 289−462) days from discharge. The mean age was 58 ± 14 (range 19−88) years, 105 (59%) were men and 158 (89%) were Caucasian. Overall 78 (44%) had one or more comorbidities (Table 1).

**Table 1:** Baseline characteristics, self‐reported symptoms and measurements at the 3 and 12 months visits (*n* = 178)

Age at discharge, years	58.2 ± 13,5	
Caucasian ethnicity	158 (89)	
Male sex	105 (59)	
Body mass index, kg/m^2^	28.1 ± 4.5	
Obesity	53 (30)	
Diabetes	14 (8)	
Hypertension	56 (32)	
Cardiovascular disease	18 (10)	
Chronic kidney disease	3 (2)	
Chronic obstructive pulmonary disease	5 (3)	
Current smoker	4 (3)	
Index hospitalization for COVID‐19		
Hospital stay, days	6 [3–11]	
Intensive care unit stay	35 (20)	
Intubated	23 (13)	
Time intubated, days	8 [5–14]	
	**3 months**	**12 months**
Dyspnea	86 (52)	84 (51)
Heart rate, per min	66 ± 11.7	67 ± 11.5
Systolic blood pressure, mmHg	134 ± 17.4	132 ± 17.4

*Note*: Continuous variables are presented as mean ± SD and median [25th−75th percentile].

#### 3.1.1 Cardiac structure and function 3 and 12 months after COVID‐19

The mean values of LV structure, LV systolic function, LV diastolic function, RV structure, and RV function were within the guideline‐recommended limits of normal at both the 3‐ and 12‐month visit (Table 2). There were no significant changes in any of the echocardiographic variables investigated from 3 to 12 months, including sensitive parameters of RV and LV function: RVLS at 3 months 26.2 ± 3.9% and at 12 months 26.5 ± 3.1%, *p* = .29; and LV GLS at 3 months 19.2 ± 2.3% and at 12 months 19.3 ± 2.3%, *p* = .64 (Table 2; Supporting Informaton: Table 2).

**Table 2:** Echocardiographic measures of left ventricular structure, systolic function and diastolic function and right ventricular structure and function at 3 months, 12 months and change from 3 to 12 months

	*N*	3 Months	12 Months	Change from 3 to 12 months		
		mean ± SD	mean ± SD	mean	95% CI	*p* Value
LVMi (g/m2)	176	68.30 ± 17.20	67.60 ± 15.90	−0.64	−1.83 to 0.52	0.27
EDVi (ml/m^2^)	166	53.57 ± 12.70	53.10 ± 12.00	0.49	−0.52 to 1.50	0.34
LVEF(%)	177	57.90 ± 5.48	58.06 ± 5.01	0.09	−0.57 to 0.39	0.72
LVGLS(%)	113	19.20 ± 2.25	19.30 ± 2.26	0.08	−0.25 to 0.41	0.64
S' (cm/s)	167	8.00 ± 1.60	8.00 ± 1.56	0.01	−0.22 to 0.24	0.93
E/A Ratio	164	1.05 ± 0.32	1.02 ± 0.36	0.03	−0.02 to 0.07	0.23
E' (cm/s)	168	8.44 ± 2.33	8.42 ± 2.20	0.03	−0.24 to 0.18	0.77
E/e’	156	8.40 ± 3.10	8.10 ± 2.60	−0.26	−0.66 to 0.14	0.20
LAVi (ml/m2)	152	26.20 ± 7.50	26.70 ± 7.90	0.49	−0.30 to 1.29	0.22
S/D ratio	131	1.36 ± 0.37	1.39 ± 0.36	0.04	−0.03 to 0.10	0.29
RVD (cm)	170	3.70 ± 0.50	3.67 ± 0.60	−0.02	−0.08 to 0.03	0.42
TAPSE (cm)	170	2.36 ± 0.32	2.34 ± 0.30	0.02	−0.01 to 0.05	0.25
RVLS(%)	122	26.20 ± 3.90	26.50 ± 3.10	0.29	−0.25 to 0.85	0.29
RVS' (cm/s)	161	13.40 ± 2.70	13.50 ± 2.75	0.17	−0.13 to 0.47	0.27
sPAP(mmHg)	102	23.7 ± 7.90	22.7 ± 9.48	−1.00	−0.80 to 2.8	0.16

Abbreviations: CI, confidence interval; E/A, E/A ratio of transmitral flow velocity; e‘, mean value of septal and lateral early diastolic pulsed tissue Doppler velocities; E/e‘, transmitral E/e'ratio; LVEDVi, left ventricular end‐diastolic volume index; LVEF, left ventricular ejection fraction; LV GLS, left ventricular global longitudinal strain; LVMi, left ventricular mass index; LV S‘, Mitral annular peak systolic velocity, cm/sec; LAVi, Left atrial volume index; PV S/D, S/D ratio of pulmonary vein; RVD, basal right ventricle diameter; RVLS, right ventricle free wall longitudinal strain; RV S‘, right ventricular peak systolic tissue Doppler velocity; SD, standard deviation; sPAP, systolic pulmonary arterial preassure; TAPSE, Tricuspid annular plane systolic excursion.

**Figure 1:**
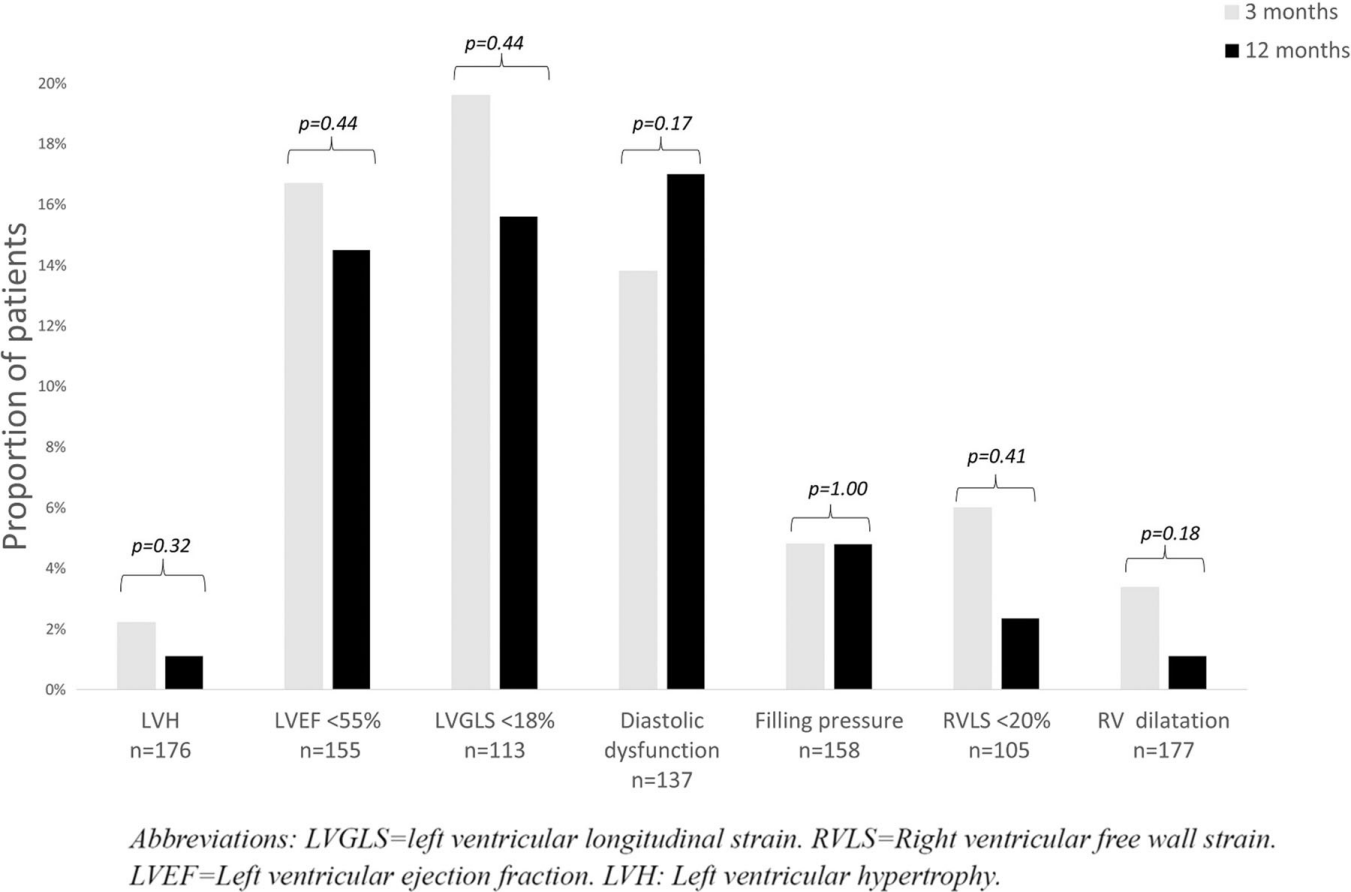
Proportion of patients with pathology on echocardiography at 3 and 12 months after COVID‐19. Proportion of patients with left ventricular (LV) hypertrophy, LV systolic dysfunction, LV diastolic dysfunction, elevated LV filling pressures, right ventricular *(*RV) dysfunction and RV dilatation. LVEF, left ventricular ejection fraction; LVGLS, left ventricular longitudinal strain; LVH, left ventricular hypertrophy; RVLS, right ventricular free wall strain.

There were no significant differences in the proportion of patients with LV hypertrophy, LVEF < 55%, LVGLS < 18%, diastolic dysfunction, increased LV filling pressures, RV dilation RVLS < 20%, and TAPSE < 1.8 cm at 3 and 12 months (Figure 1).

When assessing changes in measurements of LV and RV function by 15% improvement or deterioration from 3 to 12 months, we found that 5 patients had deterioration and 6 patients had an improvement in LVGLS (*p* = .76) (Table 3). For RVLS, 7 patients had a deterioration and 16 patients had an improvement (*p* = .06).

**Table 3:** Changes in left ventricular ejection fraction (LVEF), left ventricular longitudinal strain (LVGLS), right ventricular free wall strain (RVLS) and Tricuspid annular plane systolic excursion (TAPSE) from 3 to 12 months after COVID‐19

	10%			15%			20%		
	Deterioration	Improvement	*p* Value	Deterioration	Improvement	*p* Value	Deterioration	Improvement	*p* Value
**LVEF (*n*** = **177)**	4 (2)	10 (6)	0.11	1 (1)	5 (3)	0.10	0 (0)	2 (1)	0.15
**LVGLS (*n*** = **113)**	10 (9)	17 (15)	0.18	5 (4)	6 (5)	0.76	2 (2)	5 (4)	0.26
**RVLS (*n*** = **122)**	13 (11)	25 (21)	0.05	7 (6)	16 (13)	0.06	2 (2)	9 (7)	0.03
**TAPSE (*n*** = **170)**	22 (13)	25 (15)	0.66	6 (4)	9 (5)	0.44	2 (1)	5 (3)	0.26

*Note*: The number and proportion of patients with 10%, 15% and 20% deterioration or improvement in each echocardiographic variable, and p‐value for difference between patients with deterioration versus improvement.

Abbreviations: LVEF, left ventricular ejection fraction; LV GLS, left ventricular global longitudinal strain; RVLS, right ventricle free wall longitudinal strain; TAPSE, Tricuspid annular plane systolic excursion.

Similar findings were made in a sensitivity analysis excluding patients with known significant structural heart disease (*n* = 5).

#### 3.1.2 Changes in cardiac structure and function by COVID‐19 severity

**Figure 2:**
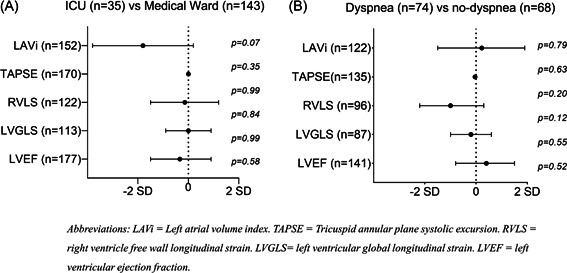
Changes in key echocardiographic parameters from 3 to 12 months stratified by COVID‐19 severity (Panel A) and dyspnea at 3 months (Panel B). Circles represent mean coefficient of the delta value and whiskers represent the corresponding 95% confidence interval from regression models. Values to the left of the X‐axis represent greater decreases in ICU‐patients (Panel A), and in patients with dyspnea (Panel B). There were no significant differences in any of the echocardiographic measures investigated As presented in Suppoting Information: Tables 2 and 3. LAVi, Left atrial volume index; LVEF, left ventricular ejection fraction; LVGLS, left ventricular global longitudinal strain; RVLS, right ventricle free wall longitudinal strain; TAPSE, Tricuspid annular plane systolic excursion.

During the index hospitalization for COVID‐19, 35 (20%) were treated in the ICU, while the remaining 143 patients were treated in the medical ward. Among patients treated in the ICU, 23 (66%) were intubated for a median of 8 days. There were no significant differences in changes in echocardiographic variables between patients treated in the ICU versus those treated in the medical ward (*p* > .05 for all; Supporting Information: Table 3; Figure 2). On admission 43 (27%) of patients were classified with a severe respiratory state during COVID‐19. There were no significant differences in changes in cardiac structure and function by respiratory state, except a larger decrease in LVMi in patients with a severe respiratory state compared to those without (−3.8 ± 8.0 vs. 0.1 ± 7.7, respectively, *p* = .007) (Supporting Information: Table 4). When comparing groups based on the presence of a severe inflammatory state during COVD‐19, there were no significant differences in changes in echocardiographic measurements between 3 and 12 months (Supporting Information: Table 5).

#### 3.1.3 Changes in cardiac structure and function by persistent dyspnea after COVID‐19

At the 3‐month visit, 74 (52%) of 142 with available data reported persistent dyspnea. At the 12‐month visit, 84 (51%) reported persistent dyspnea (98% of those with dyspnea at 3 months). There were no consistent significant differences in echocardiographic variables by dyspnea at the 3 or 12‐month visit, except for a tendency for smaller LVEDVi in patients with dyspnea at 12 months (Supporting Information: Table 6&7). There were no significant differences in changes in cardiac structure and function between patients with dyspnea at 3 months compared to those without dyspnea (*p* > .05 for all;Supporting Information: Table 8; Figure 2). The lack of association between changes in echocardiographic variables with dyspnea persisted in adjusted models (adjusted *p* > .05 for all).

### 3.2 Changes in 24‐h ECG detected arrhythmias after COVID‐19

Among patients with arrhythmia at the 3 months visit (*n* = 40), there was a nonsignificant decrease in the proportion of patients with >10% PVCs from 3 to 12 months (*n* = 30 and *n* = 21, *p* = .32) and non‐sustained ventricular tachycardia (NSVTs) (*n* = 8 and *n* = 1, *p* = .35; Supporting Information: Table 9). Patients with arrhythmia at 3 months had similar COVID‐19 severity and prevalence of symptoms as those without arrhythmia. Two patients had >1 episode of NSVT at either the 3 or 12‐month visit and both patients had severely reduced LV function with LVEF < 30%.

## 4 DISCUSSION

In this multicenter study we assessed changes in cardiac structure and function by echocardiography and arrhythmic burden by 24‐h ECG from 3 to 12 months after moderate‐to‐severe COVID‐19, and report four main findings: (1) There was no significant overall change in measures of cardiac structure and function from 3 to 12 months after COVID‐19; (2) Changes in echocardiographic parameters did not associate with COVID‐19 severity or persistent dyspnea after COVID‐19; and (3) Among patients with cardiac arrhythmias at 3 months, there were no significant changes in arrhythmic burden to 12 months. Our results therefore suggest that COVID‐19 has a limited impact on long‐term changes in cardiac structure and function and that symptoms are unlikely due to progressive cardiac dysfunction and remodeling.

Our findings may have implications for monitoring strategies post‐COVID and for a better understanding of the long‐term cardiac consequences of this disease. Our findings should be put in context of the wealth of evidence that has emerged during the last years regarding cardiac pathology from COVID‐19. It is well established that echocardiography‐detected cardiac dysfunction during the index hospitalization for COVID‐19 associates with a worse in‐hospital outcome.^6^, ^10^, ^24^ Substantial scientific efforts have been put in to understand whether this dysfunction is a result of direct cardiac damage or secondary to critical illness, pulmonary pathology and deconditioning.

The cardiac dysfunction and injury caused by moderate‐to‐severe COVID‐19 during the index hospitalization have been studied during the early convalescence period.^25^, ^26^, ^27^ Lassen et al found a reduced LV function at 3 months compared to matched controls, but with no significant change in LV GLS from hospitalization to 3‐month follow‐up. We and others have found a normal LV function in the first few months after hospitalization.^15^, ^16^, ^17^, ^28^ Gao et al. found no structural or functional cardiac pathology 12 months after COVID‐19 compared to matched controls.^29^ In the current study, we found no significant changes in any of the echocardiographic parameters from 3 to 12 months, and the proportion of patients with LV and RV dysfunction was similar at both visits. Although we did not have pre‐COVID echocardiographic data on the participants, these findings suggest that there is limited long‐term cardiac remodeling and progressive dysfunction after hospitalization for COVID‐19, and that cardiac recovery from the acute disease predominantly occurs within the first 3 months. Of note, there were numerically more patients with an improvement than deterioration in RVLS which may suggest that recovery of the RV is still ongoing after 3 months in some patients. This extends previous findings demonstrating improved RVLS from hospitalization for COVID‐19 to 3 months follow‐up.^13^ However, as the overall change in RVLS to 12 months was nonsignificant, these findings must be viewed as hypothesis‐generating.

Abnormalities in cardiac structure and function after COVID‐19 have also been investigated with the use of CMR. In these studies, the prevalence of cardiac pathology after COVID‐19 varies from 3% to 78%.^11^, ^12^, ^30^ Findings reflective of diffuse fibrosis, myocardial edema, and myocardial scar have been reported, but the clinical and prognostic impact of these findings in the absence of cardiac dysfunction is unknown. A longitudinal CMR study reported almost normalization of cardiac findings from 3 to 6 months^31^

Postacute COVID‐19 (PACS) is defined as persistent symptoms and/or delayed or long‐term complications beyond 4 weeks from the onset of COVID‐19.^32^ We found limited evidence of an association between changes in cardiac structure and function and persistent dyspnea. Increasing numbers of patients report long‐term complications and dyspnea beyond what is expected after hospitalization with COVID‐19 (i.e., PACS).^33^ In our study, 52% and 51% reported dyspnea at 3 and 12 months, respectively. Studies done in the early convalescent phase do not find any correlation between symptoms and echocardiographic findings.^13^, ^14^, ^17^, ^27^ Our study extends these findings, as we do not find an association between symptoms and changes in echocardiographic parameters between 3 and 12 months post‐COVID. There is no established pathophysiologic explanation for the persistent symptoms after COVID‐19, and our findings suggest that long‐term cardiac deterioration is unlikely to play a central role. Data from cardiopulmonary exercise testing post COVID‐19 hospitalization suggest that obesity, deconditioning, dysautonomia, and lower ventilatory efficiency may be factors that contributes to the pathophysiologic mechanisms of dyspnea in PACS.^34^, ^35^ There has also been shown a circulatory impairment,^35^ which may represent changes in pulmonary perfusion rather than cardiac dysfunction.

The role cardiac arrhythmias play after COVID‐19 is uncertain. In our study we found a nonsignificant decrease in arrhythmic burden among patients with documented arrhythmia at 3 months, and the presence of arrhythmia was not associated with COVID‐19 severity or persistent symptoms. It is unclear whether this reflects an improvement in COVID‐19 related arrhythmogenicity or if it is a result of regression to the mean as only patients *with arrhythmia* at 3 months were invited to a second recording. This exploratory finding should be investigated in future studies.

## 5 LIMITATIONS

Like most observational COVID‐19 studies, we did not have echocardiographic measurements at the index hospitalization or before hospitalization, and can therefore not assess changes in cardiac structure and function from acute COVID‐19 or pre‐COVID‐19 to the follow‐up visits. Of all patients invited to the PROLUN study, 200 (67%) had an available echocardiogram at the 3‐month visit, and among these, 178 (89%) also had an available echocardiogram at the 12‐month visit (Supporting Information: Figure 1). Although this is likely to introduce selection bias towards a healthier study population, we demonstrate comparable baseline characteristics to participants who were not part of this substudy (Supporting Information: Table 1). This study used echocardiography to assess cardiac structure and function, which is less sensitive than CMR. However, we performed more sensitive echocardiographic measurements such as myocardial strain to improve the ability to detect subtle changes. The external validity of our study to current post‐COVID‐19 care may be limited by the fact that our population was enrolled at an early stage of the pandemic with different SARS‐CoV‐2 variants and therapeutic options than today. Furthermore, Norway was mildly affected by the pandemic compared to most other countries. Therefore, the threshold for ICU treatment may have been lower than in other places. Some patients did not have available measures of all echocardiographic parameters due to image quality (i.e., 21% had missing GLS, which is better than most population‐based studies). We did not impute these variables as we investigated each echocardiographic parameter separately, and because these were the main outcome measures in the study. Due to limited capacity, only the participants with pathology at 3 months had a 24‐h ECG examination at 12 months, which introduces substantial bias. Despite multiple testing we did not adjust the *p* value as this did not have an impact on our overall neutral results.

## 6 CONCLUSIONS

Cardiac structure and function remained unchanged from 3 to 12 months after the index hospitalization for COVID‐19, irrespective of initial COVID‐19 severity. We found no association between changes in cardiac structure and function and the presence of persistent dyspnea, suggesting that these symptoms are not related to cardiac pathology from COVID‐19. There was no significant change in arrhythmic burden among patients with documented arrhythmia at 3 months. In sum, our results suggest that progression of cardiac dysfunction after COVID‐19 is rare and unlikely to play an important role in PACS.

## Supporting information

Supporting information.

## Data Availability

The data that support the findings of this study are available on request from the corresponding author. The data are not publicly available due to privacy or ethical restrictions.
