# Supplementary material for: Changes in cardiac structure and function from 3 to 12 months after hospitalization for COVID‐19
Source: Clin Cardiol. 2022 Aug 3;45(10):1044–52. doi: 10.1002/clc.23891 (PMC9538691; doi:10.1002/clc.23891)
Supplement: Supplementary file 1 — Supporting information. [file CLC-45-1044-s001.docx]

Supplemental Material

to

Changes in cardiac structure and function from 3 to 12 months after hospitalization for COVID-19

**SUPPLEMENTAL METHODS**

*Echocardiography*

Left ventricular mass was calculated from wall thickness and end-diastolic dimension, and was indexed to body surface area (BSA [m^2^]; LVMi). LV end-diastolic volume and atrial end-systolic volume were measured by the Simpson’s method in the apical four and two chamber view and indexed to BSA (LV EDVi; LAVi). LV ejection fraction (EF) was calculated using Simpson’s biplane method. Pulsed tissue Doppler systolic velocity (S’), and early diastolic velocities (e’) were obtained from the septal and lateral wall of the mitral annulus and the lateral wall of the tricuspid annulus. Ratio of the mitral valve early (E wave) and late (A wave) inflow velocities (MV E/A), ratio of E wave and e’ (E/e'), and pulmonary vein flow were assessed according to the international recommendations^1^. Motion mode was used in the apical four-chamber view to measure tricuspid annular plane systolic excursion (TAPSE). We used maximal tricuspid regurgitation velocity to estimate the systolic pulmonary artery pressure (SPAP). LV global longitudinal strain (LV GLS) and RV free wall longitudinal strain (RVLS) were quantified by semi-automatic 2D speckle tracking software (AFI) based on the apical four-, two-, and long-axis views, and the RV focus view^2^.^.^ Data were stored digitally for offline analysis (GE EchoPAC PC SWO version 204). All analyses of the echocardiograms from both visits were performed by the same investigator.

**Suppl. Table 1:** Comparison of baseline characteristics between PROLUN patients in the current echocardiography substudy and the remaining PROLUN participants.

|  | **Participants in**  **echocardiographic substudy n=178** | **Participants not in echocardiographic substudy  n=86** | **P-value** |
| --- | --- | --- | --- |
| Age at discharge, years | 58.2±13,5 | 59.2±15.7 | 0.59 |
| Caucasian ethnicity | 158 (89) | 74 (86) | 0.53 |
| Male sex | 105 (59) | 47 (55) | 0.59 |
| Body mass index, kg/m^2^ | 28.1±4.5 | 28.7±15.7 | 0.44 |
| Obesity | 53 (30) | 26 (34) | 0.48 |
| Diabetes | 14 (8) | 9 (11) | 0.49 |
| Hypertension | 56 (32) | 24 (36) | 0.61 |
| Cardiovascular disease | 18 (10) | 4 (5) | 0.13 |
| Chronic kidney disease | 3 (2) | 4 (5) | 0.16 |
| Chronic obstructive pulmonary disease | 5 (3) | 4 (5) | 0.44 |
| Current smoker | 4 (3) | 2 (3) | 0.82 |
|  |  |  |  |
| *Index hospitalization for COVID-19* | |  |  |
| Hospital stay, days | 6 [3, 11] | 6 [3, 14] | 0.77 |
| Intensive care unit stay | 35 (20) | 17 (20) | 0.98 |
| Intubated | 23 (13) | 13 (16) | 0.57 |
| Time intubated, days | 8 [7, 14] | 12 [9, 15] | 0.17 |
| Heart rate, per min | 66 ±11.7 | 70.6±10.4 | 0.08 |
| Systolic blood pressure, mm Hg | 134 ±17.4 | 133±20.8 | 0.42 |

**Suppl. Table 2:** Echocardiographic measures of left ventricular structure, systolic function and diastolic function and right ventricular structure and function at 3 months, 12 months and change from 3 to 12 months. Compared with Wilcoxon matched-pairs signed-rank test.

|  | N | 3 Months | 12 Months | Change from 3 to 12 months | | |
| --- | --- | --- | --- | --- | --- | --- |
|  |  | *Median* | *median* | *Median* | [Q1-Q3] | *P-value* |
| LVMi (g/m2) | 176 | 65.8[57,76] | 64.3[56.7, 77.5] | -0.60 | [-3.87, 3.0] | 0.32 |
| LVEF(%) | 177 | 59[56, 61] | 58[56, 61] | 0 | [-2, 2] | 0.87 |
| LVGLS(%) | 113 | 19[18, 21] | 19[18, 20.9] | 0 | [-1, 1] | 0.74 |
| E/A Ratio | 164 | 1[0.8, 1.2] | 1[0.8,1.2] | 0.02 | [-0.1, 0.2] | 0.01 |
| LAVi (ml/m2) | 152 | 28[24, 31] | 25[20.7, 30] | 0.4 | [-2.4, 2.5] | 0.32 |
| RVD (cm) | 170 | 3.7[3.4, 4] | 3.6[3.3, 4] | 0 | [-0.2, 0.2] | 0.27 |
| RVLS(%) | 122 | 26[23.5, 28.3] | 26[24, 28] | 0 | [-1.3, 2] | 0.37 |
| RVS´ (cm/s) | 161 | 13[11.3, 15] | 13[11.7, 15.4] | 0 | [-0.9, 1.2] | 0.39 |
| sPAP(mmHg) | 102 | 23[18, 28] | 22.3[14.2, 26.6] | -1.8 | [-8.1, 3.7] | 0.16 |

**Abbreviations**: LVMi = left ventricular mass index; LVEF = left ventricular ejection fraction; LV GLS= left ventricular global longitudinal strain; E/A= E/A ratio of transmitral flow velocity; LAVi = Left atrial volume index; RVD = basal right ventricle diameter; RVLS = right ventricle free wall longitudinal strain; RVS` = right ventricular peak systolic tissue Doppler velocity. sPAP=  systolic pulmonary arterial pressure

**Suppl. Table 3:** Changes in cardiac structure and function (mean ± SD) from 3 to 12 months in COVID-19 patients treated in the ICU compared to the medical ward, and p-value for the difference in change by univariable and multivariable linear regression.

|  | Medical Ward | ICU | P-value | Adjusted P-value |
| --- | --- | --- | --- | --- |
|  | **n=143** | **n=35** |  |  |
|  |  |  |  |  |

| Δ **LVMi**  (g/m2) | -0.8 ± 8.3 | -0.1 ± 6.2 | 0.75 | 0.87 |
| --- | --- | --- | --- | --- |
| **Δ EDVi** (ml/m^2^) | -0.4 ± 6.8 | -0.8 ± 5.8 | 0.75 | 0.27 |
| **Δ LVEF** (%) | 0.2 ± 3.3 | -0.2 ± 3.0 | 0.58 | 0.20 |
| **Δ LVGLS** (%) | 0.1 ± 1.9 | 0.1 ± 1.3 | 0.99 | 0.97 |
| **Δ S'** (cm/s) | 0.0 ± 1.5 | -0.2 ± 1.5 | 0.38 | 0.30 |
| **Δ E/A Ratio** | 0.0 ± 0.3 | 0.1 ± 0.2 | 0.10 | 0.06 |
| **Δ E'**(cm/s) | -0.1 ± 1.4 | 0.1 ± 1.2 | 0.56 | 0.98 |
| **Δ E/e'** | -0.2 ± 2.6 | -0.5 ± 2.0 | 0.58 | 0.89 |
| **Δ RV GLS**(%) | 0.3 ± 3.0 | 0.2 ± 3.3 | 0.84 | 0.81 |
| **Δ RVS´**(cm/s) | 0.1 ± 1.9 | 0.5 ± 1.8 | 0.22 | 0.14 |
| **Δ RVD**(cm) | -0.0 ± 0.4 | -0.0 ± 0.3 | 0.81 | 0.97 |
| **Δ TAPSE**(cm) | 0.0 ± 0.2 | 0.0 ± 0.2 | 0.99 | 0.28 |
| **Δ sPAP** (mmHg) | -3.4 ± 7.8 | -1.0 ± 9.6 | 0.31 | 0.41 |
| **Δ LAVi** (ml/m2) | 0.8 ± 5.2 | -1.0 ± 3.4 | 0.07 | 0.17 |
| **Δ S/D ratio** | 0.0 ± 0.4 | 0.1 ± 0.4 | 0.42 | 0.25 |

**Abbreviations**: LVMi = left ventricular mass index; LVEDVi = left ventricular end-diastolic volume index; LVEF = left ventricular ejection fraction; LV GLS= left ventricular global longitudinal strain; LV S`; Mitral annular peak systolic velocity, cm/sec; E/A= E/A ratio of transmitral flow velocity; e`= mean value of septal and lateral early diastolic pulsed tissue Doppler velocities ; E/e`= transmitral E/e`ratio; LAVi = Left atrial volume index; PV S/D = S/D ratio of pulmonary vein; RVD = basal right ventricle diameter; TAPSE = Tricuspid annular plane systolic excursion; RVLS = right ventricle free wall longitudinal strain; RV S` = right ventricular peak systolic tissue Doppler velocity. sPAP=  systolic pulmonary arterial pressure

* adjusted for age, sex, systolic blood pressure, heart rate, BMI and established cardiovascular disease, in addition to the baseline echocardiographic value (3 month visit).

**Suppl. Table 4:** Changes in cardiac structure and function (mean ± SD) from 3 to 12 months in COVID-19 patients with severe vs non-severe COVID-19 at hospital admission based on respiratory vital signs. P-value for the difference in change by univariable and multivariable linear regression.

|  | Severe COVID-19  n=113 | Non-Severe COVID-19 | P-value | Adjusted P-value |
| --- | --- | --- | --- | --- |
|  |  | **n=43** |  |  |
|  |  |  |  |  |

| **Δ LVMi (g/m2)** | -0.2 ± 0.8 | -3.7 ± 1.2 | 0.02 | 0.03 |
| --- | --- | --- | --- | --- |
| **Δ EDVi (ml/m^2^)** | -0.2 ± 0.7 | -1.8 ± 1.1 | 0.21 | 0.09 |
| **Δ LVEF (%)** | 0.2 ± 0.3 | -0.6 ± 0.5 | 0.20 | 0.51 |
| **Δ LVGLS (%)** | -0.0 ± 0.2 | 0.3 ± 0.4 | 0.49 | 0.96 |
| **Δ S' (cm/s)** | -0.1 ± 0.2 | 0.4 ± 0.3 | 0.10 | 0.28 |
| **Δ E/A Ratio** | 0.0 ± 0.0 | -0.0 ± 0.0 | 0.45 | 0.17 |
| **Δ E'(cm/s)** | -0.1 ± 0.1 | 0.0 ± 0.2 | 0.70 | 0.78 |
| **Δ E/e'** | -0.2 ± 0.3 | -0.6 ± 0.4 | 0.45 | 0.49 |
| **Δ RV GLS(%)** | 0.6 ± 0.4 | -0.5 ± 0.5 | 0.10 | 0.32 |
| **Δ RVS´(cm/s)** | 0.1 ± 0.2 | 0.6 ± 0.3 | 0.18 | 0.22 |
| **Δ RVD(cm)** | -0.0 ± 0.0 | -0.0 ± 0.1 | 0.92 | 0.47 |
| **Δ TAPSE(cm)** | 0.0 ± 0.0 | 0.0 ± 0.0 | 0.48 | 0.40 |
| **Δ sPAP (mmHg)** | -0.0 ± 0.1 | 0.1 ± 0.1 | 0.25 | 0.30 |
| **Δ LAVi (ml/m2)** | 0.9 ± 0.5 | -0.5 ± 0.9 | 0.18 | 0.09 |
| **Δ S/D ratio** | 0.0 ± 0.0 | 0.1 ± 0.1 | 0.25 | 0.28 |

**Abbreviations**: LVMi = left ventricular mass index; EDVi = left ventricular end-diastolic volume index; LVEF = left ventricular ejection fraction; LV GLS= left ventricular global longitudinal strain; LV S`; Mitral annular peak systolic velocity, cm/sec; E/A= E/A ratio of transmitral flow velocity; e`= mean value of septal and lateral early diastolic pulsed tissue Doppler velocities ; E/e`= transmitral E / e`ratio; LAVi = Left atrial volume index; PV S/D = S/D ratio of pulmonary vein; RVD = basal right ventricle diameter; TAPSE = Tricuspid annular plane systolic excursion; RVLS = right ventricle free wall longitudinal strain; RV S` = right ventricular peak systolic tissue Doppler velocity. sPAP=  systolic pulmonary arterial pressure

* adjusted for age, sex, systolic blood pressure, heart rate, BMI and established cardiovascular disease, in addition to the baseline echocardiographic value (3 month visit).

**Suppl. Table 5:** Changes in cardiac structure and function (mean ± SD) from 3 to 12 months in COVID-19 patients with above and below the median CRP level at admission. P-value for the difference in change by univariable and multivariable linear regression

|  | CRP>113 mg/L  n=88 | CRP<113 mg/L  N=87 | P-value | Adjusted P-value |
| --- | --- | --- | --- | --- |
|  |  |  |  |  |

| **Δ LVMi (g/m2)** | 0.1 ± 7.7 | -1.4 ± 8.1 | 0.22 | 0.19 |
| --- | --- | --- | --- | --- |
| **Δ EDVi (ml/m^2^)** | -1.0 ± 6.7 | 0.0 ± 6.5 | 0.30 | 0.82 |
| **Δ LVEF (%)** | 0.1 ± 3.1 | -0.0 ± 3.3 | 0.84 | 0.85 |
| **Δ LVGLS (%)** | 0.2 ± 1.6 | -0.1 ± 2.0 | 0.50 | 0.40 |
| **Δ S' (cm/s)** | -0.0 ± 1.6 | -0.1 ± 1.4 | 0.89 | 0.86 |
| **Δ E/A Ratio** | 0.0 ± 0.2 | 0.0 ± 0.4 | 0.56 | 0.83 |
| **Δ E'(cm/s)** | -0.1 ± 1.5 | -0.1 ± 1.3 | 1.00 | 0.89 |
| **Δ E/e'** | -0.6 ± 2.5 | 0.1 ± 2.5 | 0.13 | 0.12 |
| **Δ RV GLS(%)** | 0.2 ± 2.6 | 0.4 ± 3.5 | 0.79 | 0.60 |
| **Δ RVS´(cm/s)** | 0.1 ± 1.9 | 0.2 ± 1.9 | 0.75 | 0.46 |
| **Δ RVD(cm)** | -0.0 ± 0.4 | -0.0 ± 0.3 | 0.80 | 0.77 |
| **Δ TAPSE(cm)** | 0.0 ± 0.2 | 0.0 ± 0.2 | 0.80 | 0.58 |
| **Δ sPAP (mmHg)** | 0.0 ± 0.5 | 0.0 ± 0.5 | 0.84 | 0.36 |
| **Δ LAVi (ml/m2)** | 0.9 ± 5.2 | 0.1 ± 4.8 | 0.34 | 0.25 |
| **Δ S/D ratio** | 0.1 ± 0.4 | 0.0 ± 0.4 | 0.71 | 0.98 |

**Abbreviations**: LVMi = left ventricular mass index; EDVi = left ventricular end-diastolic volume index; LVEF = left ventricular ejection fraction; LV GLS= left ventricular global longitudinal strain; LV S`; Mitral annular peak systolic velocity, cm/sec; E/A= E/A ratio of transmitral flow velocity; e`= mean value of septal and lateral early diastolic pulsed tissue Doppler velocities ; E/e`= transmitral E / e`ratio; LAVi = Left atrial volume index; PV S/D = S/D ratio of pulmonary vein; RVD = basal right ventricle diameter; TAPSE = Tricuspid annular plane systolic excursion; RVLS = right ventricle free wall longitudinal strain; RV S` = right ventricular peak systolic tissue Doppler velocity. sPAP=  systolic pulmonary arterial pressure

* adjusted for age, sex, systolic blood pressure, heart rate, BMI and established cardiovascular disease, in addition to the baseline echocardiographic value (3 month visit).

**Suppl. Table 6*:*** Echocardiographic measurements in patients with, and without dyspnea at 3 months

|  | **No Dyspnea** | **Dyspnea** | **P-value** |
| --- | --- | --- | --- |
|  | **n=68** | **n=74** |  |
| **LVMi**  (g/m2) | 67.1±16.5 | 68.2 ± 18.0 | 0.71 |
| **EDVi** (ml/m^2^) | 54.7 ± 13.5 | 50.7 ± 10.4 | 0.05 |
| **LVEF** (%) | 58.3 ± 4.5 | 57.5 ± 6.1 | 0.38 |
| **LVGLS** (%) | 19.1 ± 2.6 | 19.3 ± 1.9 | 0.68 |
| **S'** (cm/s) | 8.1 ± 1.4 | 7.9 ± 1.7 | 0.35 |
| **E/A Ratio** | 1.0 ± 0.3 | 1.0 ± 0.2 | 0.50 |
| **Δ E'**(cm/s) | 8.8 ± 2.3 | 8.3 ± 2.3 | 0.16 |
| **E/e'** | 7.8 ± 3.0 | 8.6 ± 3.0 | 0.12 |
| **RV GLS** (%) | 25.8 ± 3.1 | 25.9 ± 4.6 | 0.89 |
| **RVS´** (cm/s) | 13.4 ± 3.0 | 13.3 ± 2.6 | 0.95 |
| **RVD** (cm) | 3.7 ± 0.5 | 3.7 ± 0.6 | 0.74 |
| **TAPSE** (cm) | 2.4 ± 0.4 | 2.3 ± 0.3 | 0.46 |
| **sPAP** (mmHg) | 23.0 ± 7.5 | 22.6 ± 7.6 | 0.78 |
| **LAVi** (ml/m2) | 25.1 ± 7.8 | 25.7 ± 7.0 | 0.64 |
| **S/D ratio** | 1.3 ± 0.3 | 1.3 ± 0.3 | 0.54 |

**Abbreviations**: LVMi = left ventricular mass index; EDVi = left ventricular end-diastolic volume index; LVEF = left ventricular ejection fraction; LV GLS= left ventricular global longitudinal strain; LV S`; Mitral annular peak systolic velocity, cm/sec; E/A= E/A ratio of transmitral flow velocity; e`= mean value of septal and lateral early diastolic pulsed tissue Doppler velocities ; E/e`= transmitral E / e`ratio; LAVi = Left atrial volume index; PV S/D = S/D ratio of pulmonary vein; RVD = basal right ventricle diameter; TAPSE = Tricuspid annular plane systolic excursion; RVLS = right ventricle free wall longitudinal strain; RV S` = right ventricular peak systolic tissue Doppler velocity. sPAP=  systolic pulmonary arterial pressure

**Suppl. Table 7*:*** Echocardiographic measurements in patients with and without dyspnea at 12 months.

|  | **No Dyspnea** | **Dyspnea** | **P-value** |
| --- | --- | --- | --- |
|  | **n=80** | **n=84** |  |
| **LVMi**  (g/m2) | 69.4±15.3 | 66.0±16.9 | 0.18 |
| **EDVi** (ml/m^2^) | 56.1±12.8 | 52.0±11.1 | 0.03 |
| **LVEF** (%) | 57.3±5.4 | 58.3±5.5 | 0.28 |
| **LVGLS** (%) | 19.0±2.5 | 19.4±1.9 | 0.28 |
| **S'** (cm/s) | 8.3±1.8 | 7.7±1.4 | 0.02 |
| **E/A Ratio** | 1.0±0.3 | 1.0±0.2 | 0.79 |
| **E'**(cm/s) | 8.9±2.2 | 8.2±2.3 | 0.05 |
| **E/e'** | 7.9 ± 3.2 | 8.4 ± 2.4 | 0.24 |
| **RV GLS** (%) | 26.3±3.6 | 25.4±4.5 | 0.18 |
| **RVS´** (cm/s) | 13.6±3.1 | 12.2±0.6 | 0.38 |
| **RVD** (cm) | 3.8±0.5 | 3.6±0.6 | 0.16 |
| **TAPSE** (cm) | 2.4 ± 0.3 | 2.3 ± 0.3 | 0.12 |
| **sPAP** (mmHg) | 22.6±8.5 | 23.3±7.0 | 0.62 |
| **LAVi** (ml/m2) | 26.5±8.1 | 25.5±6.9 | 0.41 |
| **S/D ratio** | 1.4±0.3 | 1.3±0.3 | 0.54 |

**Abbreviations**: LVMi = left ventricular mass index; LVEDVi = left ventricular end-diastolic volume index; LVEF = left ventricular ejection fraction; LV GLS= left ventricular global longitudinal strain; LV S`; Mitral annular peak systolic velocity, cm/sec; E/A= E/A ratio of transmitral flow velocity; e`= mean value of septal and lateral early diastolic pulsed tissue Doppler velocities ; E/e`= transmitral E / e`ratio; LAVi = Left atrial volume index; PV S/D = S/D ratio of pulmonary vein; RVD = basal right ventricle diameter; TAPSE = Tricuspid annular plane systolic excursion; RVLS = right ventricle free wall longitudinal strain; RV S` = right ventricular peak systolic tissue Doppler velocity. sPAP=  systolic pulmonary arterial pressure

**Suppl. Table 8:** Changes on cardiac structure and function (mean ± SD) from 3 to 12 months in COVID-19 patients with and without dyspnea at 3 months, and p-value for the difference in change by univariable and multivariable linear regression.

|  | No Dyspnea n=68 | Dyspnea n=74 | P-value | Adjusted P-value |
| --- | --- | --- | --- | --- |

| Δ **LVMi**  (g/m2) | -1.2± 7.0 | -0.9±8.2 | 0.86 | 0.81 |
| --- | --- | --- | --- | --- |
| **Δ EDVi** (ml/m^2^) | -0.6± 6.4 | -0.3±6.4 | 0.79 | 0.17 |
| **Δ LVEF** (%) | -0.0± 3.1 | 0.3±3.8 | 0.52 | 0.26 |
| **Δ LVGLS** (%) | 0.2± 1.8 | -0.1±1.9 | 0.55 | 0.71 |
| **Δ S'** (cm/s) | -0.2± 1.6 | 0.1±1.5 | 0.31 | 0.51 |
| **Δ E/A Ratio** | 0.0± 0.3 | 0.1±0.2 | 0.43 | 0.50 |
| **Δ E'**(cm/s) | -0.1± 1.4 | 0.1±1.4 | 0.27 | 0.52 |
| **Δ E/e'** | -0.1± 2.6 | -0.4±2.4 | 0.48 | 0.91 |
| **Δ RV GLS**(%) | 0.6± 3.4 | -0.4±2.4 | 0.12 | 0.30 |
| **Δ RVS´**(cm/s) | 0.4± 2.1 | -0.1±1.9 | 0.20 | 0.55 |
| **Δ RVD**(cm) | 0.0± 0.3 | -0.1±0.4 | 0.09 | 0.10 |
| **Δ TAPSE**(cm) | 0.0± 0.2 | 0.0±0.2 | 0.20 | 0.22 |
| **Δ sPAP** (mmHg) | -1.1± 7.8 | -0.0±10.5 | 0.66 | 0.67 |
| **Δ LAVi** (ml/m2) | 0.1± 4.6 | 0.4±4.9 | 0.79 | 0.87 |
| **Δ S/D ratio** | 0.1± 0.4 | 0.1±0.4 | 0.62 | 0.80 |

**Abbreviations**: LVMi = left ventricular mass index; LVEDVi = left ventricular end-diastolic volume index; LVEF = left ventricular ejection fraction; LV GLS= left ventricular global longitudinal strain; LV S`; Mitral annular peak systolic velocity, cm/sec; E/A= E/A ratio of transmitral flow velocity; e`= mean value of septal and lateral early diastolic pulsed tissue Doppler velocities ; E/e`= transmitral E / e`ratio; LAVi = Left atrial volume index; PV S/D = S/D ratio of pulmonary vein; RVD = basal right ventricle diameter; TAPSE = Tricuspid annular plane systolic excursion; RVLS = right ventricle free wall longitudinal strain; RV S` = right ventricular peak systolic tissue Doppler velocity; sPAP=  systolic pulmonary arterial pressure.

* adjusted for age, sex, systolic blood pressure, heart rate, BMI and established cardiovascular disease, in addition to the baseline echocardiographic value (3 month visit).

**Suppl.Table 9:** Arrhythmias recorded on 24-h ECG at 3 and 12 months. Only patients with abnormal 24-h ECG at the 3 month visit had a second 24-h ECG at the 12 month visit, and were included in this subgroup analysis (n=40).

|  | 3 months | 12 months | *P-value* |
| --- | --- | --- | --- |
| Non-sustained ventricular tachycardia | 8 (20%) | 1 (3%) | *0.35* |
| >1 episode of non-sustained ventricular tachycardia | 1 (3%) | 1 (3%) | *1.00* |
| Premature ventricular contractions >10% / 24 hour | 1 (3%) | 2 (5%) | *0.32* |
| Atrial fibrillation/flutter | 3 (5%) | 1 (3%) | *0.41* |
| Second or third degree atrioventricular block | 0 | 1 (3%) | *0.32* |
| Extreme sinus bradycardia (<30 bpm) | 0 | 1 (3%) | *0.32* |
| Sinoatrial block >3 seconds | 1 (3%) | 2 (5%) | *0.37* |
| Supraventricular tachycardia >30 seconds | 1 (3%) | 1 (3%) | *1.00* |

**Suppl.Figure 1:**CONSORT flow diagram of the PROLUN study

**
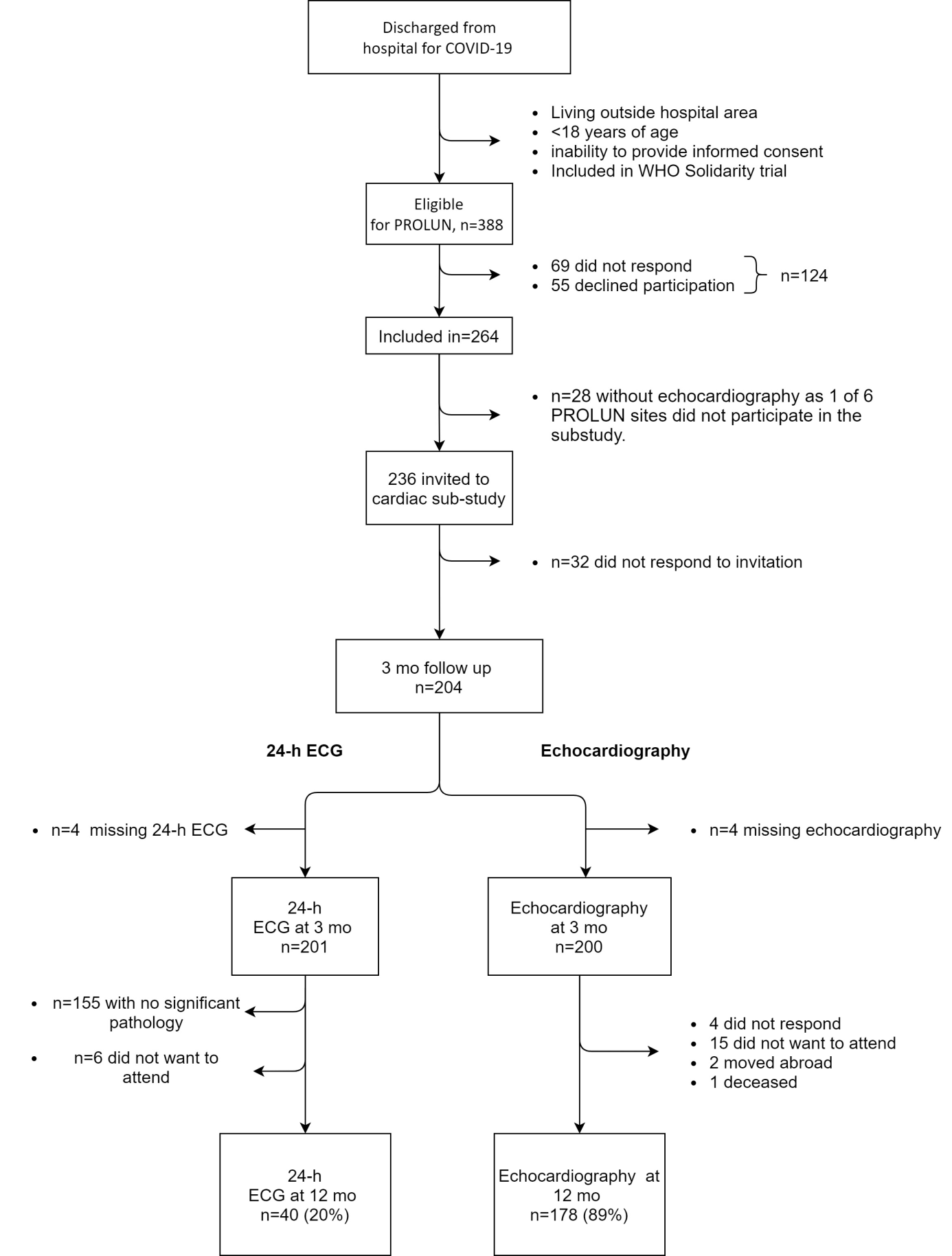
**

**Supplemental references:**

1. Recommendations for Cardiac Chamber Quantification by Echocardiography in Adults: An Update from the American Society of Echocardiography and the European Association of, Cardiovascular Imaging. *Eur Heart J Cardiovasc Imaging*. 2016;17:412-412. doi: 10.1093/ehjci/jew041

2. Mitchell C, Rahko PS, Blauwet LA, Canaday B, Finstuen JA, Foster MC, Horton K, Ogunyankin KO, Palma RA, Velazquez EJ. Guidelines for Performing a Comprehensive Transthoracic Echocardiographic Examination in Adults: Recommendations from the American Society of Echocardiography. *J Am Soc Echocardiogr*. 2019;32:1-64. doi: 10.1016/j.echo.2018.06.004
